# Supplementary material for: Effects of Humic Acids on the Ecotoxicity of Fe3O4 Nanoparticles and Fe-Ions: Impact of Oxidation and Aging
Source: Nanomaterials (Basel). 2020 Oct 12;10(10):2011. doi: 10.3390/nano10102011 (PMC7600131; doi:10.3390/nano10102011)
Supplement: Supplementary file 1 [file nanomaterials-10-02011-s001.pdf]

nanomaterials

MDPI

Supplementary Materials

Effects of Humic Acids on the Ecotoxicity of Fe_3O_4 Nanoparticles and Fe-Ions: Impact of Oxidation and Aging

Lyubov Bondarenko [1,*], Anne Kahru [2], Vera Terekhova [3,5], Gulzhian Dzhardimalieva [1,4], Pavel Uchanov [5] and Kamila Kydralieva [1]

[1] Moscow Aviation Institute, National Research University, 125993 Moscow, Russia; dzhardim@icp.ac.ru (G.D.); KydralievaKA@mai.ru (K.K.)
[2] Laboratory of Environmental Toxicology, National Institute of Chemical Physics and Biophysics, 12618 Tallinn, Estonia; anne.kahru@kbfi.ee
[3] Laboratory of Ecotoxicological Analysis of Soil, Faculty of Soil Science, Lomonosov Moscow State University, 119991 Moscow, Russia; vterekhova@gmail.com
[4] Laboratory of Metallopolymers, Polymers and Composite Materials Department, Institute of Problems of Chemical Physics RAS, 142432 Chernogolovka, Moscow region, Russia
[5] The Laboratory of Ecological Functions of Soil, Institute of Ecology and Evolution, RAS, 119071 Moscow, Russia; p.uchanov@gmail.com
* Correspondence: BondarenkoLS@mai.ru Tel.: +7-901-743-3454

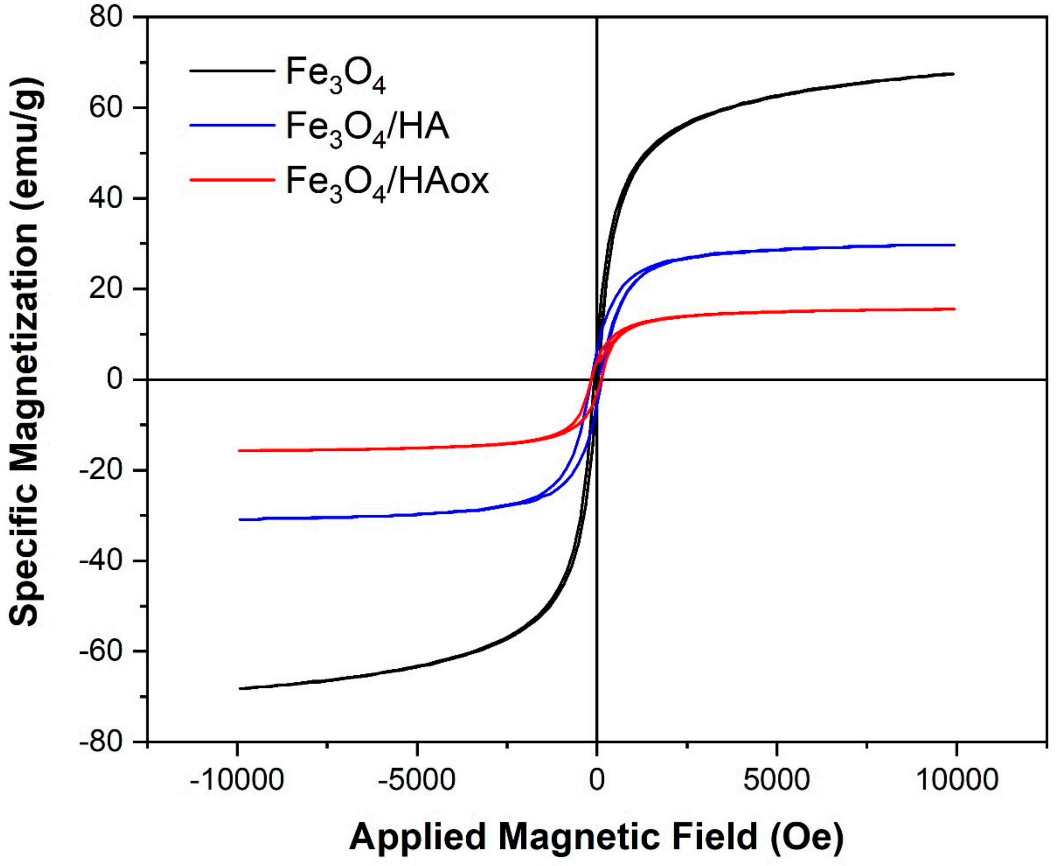
Fe3O4
Fe3O4/HA
Fe3O4/HAox
Specific Magnetization (emu/g)
Applied Magnetic Field (Oe)
80
60
40
20
0
-20
-40
-60
-80
-10000
-5000
0
5000
10000

Figure S1.

Table S1. Exposure concentration (mg Fe/L) and concentration for soluble Fe-salt and studied MNPs (mg/L, without color).

**Samples**	**Ciliates**							
	**0.25%**	**0.05%**	**0.025%**	**0.005%**	**0.0025%**	**0.0005%**	**0.00025%**	**0.00005%**
Fe^{2+}	745	149	74.5	14.9	7.45	1.49	0.745	0.149
Fe^{3+}	770	154	77	15.4	7.7	1.54	0.77	0.154
$FeCl_2$, $FeCl_3$	1650	330	165	33	16.5	3.3	1.65	0.33
Fe^{2+}/Fe^{3+}	-	-	-	15.1	7.55	1.51	0.76	0.151
$FeCl_2/FeCl_3$				33	16.5	3.3	1.65	0.33
Fe(II)HA	-	-	-	14.9	7.45	1.49	0.745	0.149
Fe(III)HA	-	-	-	15.4	7.7	1.54	0.77	0.154
Fe(II,III)HA	-	-	-	15.1	7.55	1.51	0.76	0.151
Salt with HA	-	-	-	33	16.5	3.3	1.65	0.33
Fe^{2+}/Fe^{3+} (90 days)	755	151	75.5	15.1	7.55	1.51	0.76	0.151
Fe(II,III)HA (90 days)	755	151	75.5	15.1	7.55	1.51	0.76	0.151
$FeCl_2/FeCl_3$ (90 days), $FeCl_2/FeCl_3$/HA (90 days)	1650	330	165	33	16.5	3.3	1.65	0.33
Fe_3O_4, Fe_3O_4/HA, Fe_3O_4 (90 days), Fe_3O_4/HA (90 days), Fe_3O_4/HAox	-	-	-	33	16.5	3.3	1.65	0.33
HA, HA (90 days)	-	-	-	33	16.5	3.3	1.65	-

Samples	**The higher plants**					
	**0.5%**	**0.25%**	**0.05%**	**0.025%**	**0.005%**	**0.0005%**
Fe^{2+}	1490	745	149	74.5	14.9	1.49
Fe^{3+}	1540	770	154	77	15.4	1.54
Fe^{2+}/Fe^{3+}	1510	755	151	75.5	15.1	1.51
Fe(II)HA	1490	745	149	74.5	14.9	1.49
Fe(III)HA	1540	770	154	77	15.4	1.54
Fe(II,III)HA	1510	755	151	75.5	15.1	1.51
Fe^{2+}/Fe^{3+} (90 days), Fe(II,III)HA (90 days)	1510	755	151	75.5	15.1	1.51
Salts and others samples	3300	1650	330	165	33	16.5

Table S2. p-Values calculated with R using ANOVA for the toxicity of MNPs and Fe-ions to *P. caudatum* and *S. alba.*

	p-value					
**Sample**	**concentration**	**samples**				
Toxicity to *P. caudatum (mg/L)*						
Fe^{2+}	0.0001	Fe^{2+}	>0.05		Fe^{2+}	
Fe^{3+}	0.0001	Fe^{3+}		0.0001	HA	0.0001
Fe^{2+}/Fe^{3+}	>0.05	Fe^{2+}/Fe^{3+}			Fe(II)HA	
Fe_3O_4	0.001	Fe_3O_4			Fe^{3+}	
HA	0.01	Fe_3O_4			HA	0.0001
Fe(II)HA	0.001	HA		0.01	Fe(III)HA	
Fe(III)HA	0.001	Fe_3O_4/HA			Fe^{2+}/Fe^{3+}	
Fe(II,III)HA	>0.05	Fe(II,III)HA		>0.05	HA	0.0001
Fe_3O_4/HA	0.0001	Fe_3O_4/HA			Fe(II,III)HA	
Toxicity to *S. alba (mg/L)*						
Fe^{2+}	0.0001	Fe^{2+}	0.001		Fe^{2+}	0.0001
Fe^{3+}	0.0001	Fe^{3+}		0.0001	HA	
Fe^{2+}/Fe^{3+}	0.0001	Fe^{2+}/Fe^{3+}			Fe(II)HA	
Fe_3O_4	>0.05	Fe_3O_4			Fe^{3+}	
HA	0.0001	Fe_3O_4		0.0001	HA	0.0001
HA/Fe^{2+}	0.001	HA			HA/Fe^{3+}	
HA/Fe^{3+}	0.0001	Fe_3O_4/HA			$Fe^{2+/3+}$	
Fe(II,III)HA	0.0001	HA/$Fe^{2+/3+}$		0.01	HA	0.0001
Fe_3O_4/HA	0.0001	Fe_3O_4/HA			Fe(II,III)HA	

Table S3. p-Values for the toxicity (EC50) of humic acids, as prepared and aged Fe^{2+}/Fe^{3+} ions to *P. caudatum* and *S. alba*.

**Sample**	**p-value**	
	**concentrations**	**samples**
Toxicity to ciliates *P. caudatum* (24 h EC50)		
HA*	0.01	0.0001
HA* (90 days)	0.001	
Fe^{2+}/Fe^{3+}	>0.05	>0.05
Fe^{2+}/Fe^{3+} (90 days)	0.0001	
Fe(II,III)HA	>0.05	0.0001
Fe(II,III)HA (90 days)	0.05	
Toxicity to plants *S. alba* (96 h EC50)		
HA*	0.0001	0.0001
HA* (90 days)	0.0001	
Fe^{2+}/Fe^{3+}	0.0001	0.001
Fe^{2+}/Fe^{3+} (90 days)	0.0001	
Fe(II,III)HA	0.0001	>0.05
Fe(II,III)HA (90 days)	0.001	
